# The Role of Adjuvant Chemotherapy before Osimertinib in Epidermal Growth Factor Receptor Mutant Resected Non-Small Cell Lung Cancer and Communicating It to Patients

**DOI:** 10.3390/curroncol31020074

**Published:** 2024-02-13

**Authors:** Paolo Maione, Rosario Salvi, Cesare Gridelli

**Affiliations:** 1 Division of Medical Oncology, S.G. Moscati Hospital, 83100 Avellino, Italy; cgridelli@libero.it; 2 Division of Thoracic Surgery, S.G. Moscati Hospital, 83100 Avellino, Italy; rosdoc@libero.it

**Keywords:** adjuvant chemotherapy, adjuvant osimertinib, EGFR mutations, NSCLC, shared decision making

## Abstract

Patients with radically resected stage II and III NSCLC are exposed to a high risk of disease recurrence. Thus, adjuvant cisplatin-based chemotherapy is routinely offered to this patient population, although it results in an absolute increase in 5-year survival rate of only 4%. This modest improvement in survival rate makes it challenging to communicate to our patients about the decision to be treated with adjuvant chemotherapy or not. Nowadays, the decision to administer adjuvant chemotherapy or not in resected NSCLC is almost never completely shared with patients because its role is very difficult to explain. The risk–benefit ratio becomes clearly unfavourable in elderly and unfit patients. Recently, the phase III ADAURA trial demonstrated a clinically significant disease-free survival and overall survival benefit with adjuvant osimertinib (with or without adjuvant chemotherapy) versus a placebo in EGFR-mutated stage IB-IIIA resected NSCLC. In this patient population, the decision to administer chemotherapy or not is much more challenging given the great benefit offered by osimertinib alone. Thus, it is time now to improve our communication tools to explain the role of adjuvant chemotherapy to our patients, especially in the EGFR-mutated population, in order to undertake real shared decision making in a clinical context in which the opportunity to administer toxic chemotherapy is debatable and subjective.

## 1. Introduction

The minority of patients with a new diagnosis of non-small cell lung cancer (NSCLC) present with resectable disease [1,2,3], and moreover, the disease recurrence rate after radical surgery, when feasible, remains unsatisfactory [4], with five-year overall survival remaining poor [5]. The poor outcomes produced by surgery alone, have stimulated over the years the search for effective adjuvant medical treatments, with adjuvant platin-based chemotherapy being the first therapeutic approach to be demonstrated to improve the survival rates of resected stage II-III NSCLC patients compared with surgery alone [6]. Several randomized phase III trials with positive results have been performed in this area, and a large meta-analysis of these trials has established that adjuvant platin-based chemotherapy provides an absolute increase in 5-year survival rate of 4% to patients with radically resected NSCLC [7]. Osimertinib, a third-generation epidermal growth factor receptor (EGFR) inhibitor, is the first targeted agent that has been demonstrated to be effective in the adjuvant setting of NSCLC. In fact, in the phase III randomized trial named ADAURA, osimertinib administered for 3 years after surgery with or without previous adjuvant chemotherapy and compared with placebo, provided a statistically significant advantage of a relevant magnitude in terms of disease-free survival (DFS) and overall survival (OS) as adjuvant treatment for patients with resected stage IB-IIIA EGFR-mutated NSCLC [8,9,10]. In the ADAURA trial, another important point was highlighted: the benefit offered by adjuvant osimertinib was independent of the previous administration of adjuvant chemotherapy, being achieved both in patients who were also administered with adjuvant chemotherapy before osimertinib and in those who were not [11]. The benefit offered by adjuvant platin-based chemotherapy is something that physicians and patients do not renounce in the majority of clinical conditions, but the magnitude of benefits is low, and a shared decision making on the choice to administer it or not is often challenging. This topic is further complicated in patients with resected EGFR-mutated NSCLC, where osimertinib alone offers a more relevant clinical benefit. Data on adjuvant chemotherapy, on adjuvant osimertinib and on communication tools with patients for real shared decision making in this clinical context are discussed in this paper.

## 2. Outcomes of Adjuvant Platin-Based Chemotherapy

### 2.1. Efficacy

In everyday clinical practice, when oncologists are faced with NSCLC patients whose tumors have been resected and diagnosed in stage II or III of disease, adjuvant chemotherapy containing cisplatin is the standard of care option they consider as a consequence of several phase III randomized trials and two relevant meta-analyses reported on this clinical context [4,7]. The LACE meta-analysis [4] which combined individual patient data from five large trials and 4584 patients with completely resected NSCLC, demonstrated a 5.4% absolute benefit in survival at five years in favour of adjuvant cisplatin-based chemotherapy, versus observation, with a reported hazard ratio of 0.89 (95% CI: 0.82–0.96). This improved efficacy outcome was demonstrated for patients whose tumors were diagnosed in stage II and IIIA of disease and was reported with several different regimen administered in the trials. One of the most studied regimens and with better outcomes in the adjuvant setting is cisplatin plus vinorelbine [12,13,14,15]. The outcomes achievable with this chemotherapeutic regimen as adjuvant strategy have also been confirmed in a meta-analysis performed on data from 1888 patients with resected NSCLC, and reporting a global advantage in survival rate at five years of 8.9% (HR = 0.8), and specifically of 4.7% in stage II and 11.6% in stage III patients [12]. In the phase III randomized trial named ANITA, 840 patients with resected stage IB-IIIA NSCLC were treated with adjuvant chemotherapy with cisplatin plus vinorelbine or observation [14]. The authors reported a clinically and statistically significant survival advantage in favour of chemotherapy with cisplatin plus vinorelbine. In fact, for patients treated with chemotherapy, a median overall survival of 65.7 months was reported, versus an overall survival of 43.7 months reported for patients who underwent only observation (HR = 0.8). In this trial, treatment with adjuvant cisplatin plus vinorelbine also produced a five-year survival rate advantage of 8.6%. Also, the JBR.10 phase III randomized trial evaluated adjuvant chemotherapy with cisplatin plus vinorelbine versus observation, but this evaluation was limited to patients with resected stage IB-II NSCLC [15]. The authors reported, also after a long-term median follow up of 9.3 years, a five-year survival rate advantage of 11% in favour of chemotherapy, but this improvement was limited to patients with stage II NSCLC (with N1 disease) with a hazard ratio of 0.68 (95% CI: 0.5–0.92). Although globally for stage IB patients treated with adjuvant chemotherapy, a survival benefit was not reported, more specifically for patients whose tumors were staged as IB but sized 4 cm or more, adjuvant chemotherapy produced an improvement in the 5-year survival rate for patients treated with chemotherapy compared with those only observed (79% versus 59% with a hazard ratio of 0.66; 95% CI, 0.39 to 1.14). Some of the most interesting data come from The International Adjuvant Lung Trial (IALT) with 1867 patients enrolled and randomized to receive three or four cycles of chemotherapy with cisplatin plus etoposide or a vinca alkaloid versus observation. For patients treated with adjuvant cisplatin-containing chemotherapy, an improved 5-year survival rate of 44.5% was reported versus 40.4% for patients randomized to observation (HR: 0.86; 95% CI: 0.76–0.98). Moreover, the improved efficacy outcomes in terms of DFS and OS were also confirmed with a long-term follow-up of 7.5 years, suggesting an eradicating effect of adjuvant chemotherapy [13]. Another very relevant meta-analysis, the Cochrane meta-analysis from the Non-Small Cell Lung Cancer Collaborative Group, was performed on individual participant data from 8447 patients and 26 randomized trials evaluating surgery plus adjuvant chemotherapy versus surgery alone. The Cochrane meta-analysis confirmed the clinically significant role of adjuvant chemotherapy, reporting for this treatment approach a 4% benefit in overall survival at five years, with a hazard ratio of 0.86 (95% CI: 0.81–0.92, *p* < 0.0001) [7].

### 2.2. Safety and Outcomes in Elderly Patients

The benefit of adjuvant chemotherapy, which is described in numerous trials, is outweighed by an unfavourable safety profile, mainly driven by cisplatin, particularly when combined with its frequently used partner in this clinical context, vinorelbine. The most relevant adverse events that have major negative impacts on quality of life are fatigue, anorexia, nausea, constipation and febrile neutropenia. Although these negative effects of adjuvant chemotherapy on quality of life are limited to the treatment period and are reversible in the majority of the cases [16,17], they are often a difficult challenge for oncologists and patients to face. Moreover, the benefit described in the above-mentioned meta-analyses, only becomes a trend toward survival advantage in elderly patients aged more than 70 years, who are, however, underrepresented in these trials [18]. In the LACE meta-analysis, this issue was addressed by reporting data from a subgroup analysis of patients aged more than 70 years. In this specific population of the meta-analysis, only a trend toward advantage in survival rate in favour of chemotherapy was reported, with a hazard ratio of 0.90 (95% CI:0.7–1.16) [4]. The results from the JBR.10 trial are only partly relevant to this topic, showing a survival benefit with adjuvant chemotherapy in an “almost elderly population”, i.e., patients aged at least 65 years (HR: 0.61; 95% CI: 0.38–0.98), who are different from a real elderly population [18]. In clinical practice, elderly patients aged 70–75 without relevant comorbidities, are often treated with sub-optimal adjuvant chemotherapy (carboplatin-based chemotherapy or cisplatin-based chemotherapy with cisplatin given at reduced dosage). Elderly patients aged 75–80 and without relevant comorbidities are, in contrast, often excluded from adjuvant chemotherapy, while a minority are administered carboplatin-based chemotherapy with carboplatin at a reduced dosage. Elderly patients aged more than 80 years, even if without relevant comorbidities, are almost never treated with adjuvant chemotherapy.

## 3. Shared Decision Making When Administering Adjuvant Chemotherapy to Patients Whose Tumors Do Not Harbour EGFR Mutation

When deciding if to administer chemotherapy and as a consequence, when sharing with our cancer patients the risk–benefit ratio of administering adjuvant chemotherapy, two main factors have to be considered: the absolute increase in the probability of survival (or the absolute reduction in the risk of death) when receiving chemotherapy, and the probability of administering toxic chemotherapy to an already surgery-cured patient. The first factor depends only on the outcomes of chemotherapy, the second also on the stage of disease and other prognostic factors of each individual patient. We must remember that the absolute risk reduction (ARR) is the inverse of the number needed to treat (NNT) and that the NNT is the number of patients that we have to treat to achieve the desired outcome (in this clinical context, patient alive at 5 years) with a certain treatment (adjuvant chemotherapy) [19,20]. The absolute increase in survival rate (or absolute risk reduction in death) of adjuvant chemotherapy versus observation is 4% at 5 years [7]. This means that the number needed to treat with adjuvant chemotherapy to save one life is 25 patients. This also means that we have to treat 100 patients with toxic chemotherapy to save four lives and that we administer toxic chemotherapy that does not achieve the aim of eradicating disease/saving life to 96 of 100 patients. However, if we are deciding on a stage III patient, we have the probability of 75% (recurrence rate) of administering chemotherapy to an already ill patient, and only a 25% probability of administering toxic chemotherapy to an already surgery-cured patient. Among these 75/100 patients, we will save 4–5 patients. However, we will give a type of first-line chemotherapy to the remaining 70 patients, prolonging the survival of a proportion of them. When deciding treatment for a stage II patient, the number needed to treat remains 25, but with about a 50% probability of administering toxic chemotherapy to an already cured patient, and a probability of about 50% of administering a type of “first-line chemotherapy” to an already metastatic patient. In other words, in a stage II patient, we will administer about 50 completely ineffective and toxic chemotherapy treatments for every 100 administered. However, the emotional and psychological status of patients in the perioperative period, and specifically when deciding on adjuvant chemotherapy, also has to be considered. A very interesting recent survey reported data on psychological states regarding the adjuvant chemotherapy of 101 patients who underwent complete surgical resection of stage II or III NSCLC and received information on postoperative adjuvants, with the data being collected by a self-administered online questionnaire [21]. The great majority of the patients included in the survey (more than 75%) answered that were very worried about the possibility of recurrence of lung cancer, even if radically resected, and as a consequence, 70% of patients chose the option “I will get adjuvant chemotherapy on any precondition” and clearly expressed the will “to do everything I can do now to prevent recurrence”. It must be underlined that the majority of patients included in this survey were aged less than 70 years (about 70%), making these psychological considerations not generalizable to elderly patients who have particular psychological reactions to cancer disease.

## 4. Outcomes of Adjuvant Osimertinib

### 4.1. Efficacy

Adjuvant osimertinib has been recently demonstrated in the phase III randomized trial named ADAURA to significantly improve disease-free survival of patients with resected EGFR-mutated stage IB-IIIA NSCLC, compared with a placebo. This outcome was achieved in patients both treated or not treated with previous adjuvant chemotherapy [8,10]. Moreover, very recently, the outcomes in terms of overall survival from the ADAURA trial were also reported, and these outcomes demonstrate an improvement in the five-year survival rate with the strategy of adjuvant osimertinib [9]. In the ADAURA trial, 682 patients were randomized to receive, in a 1:1 ratio, osimertinib (80 mg once daily) or placebo for 3 years unless there was disease recurrence or occurrence of severe toxicity. The primary end point was disease-free survival among patients with stage II to IIIA disease, while secondary end points included disease-free survival among patients with stage IB to IIIA disease, overall survival, and safety. Of 339 patients who received osimertinib, 66% of patients completed the planned three years of treatment, and 41% of the 343 patients who received a placebo also reached the endpoint. As for the primary endpoint, disease-free survival in patients with stage II-IIIA, osimertinib achieved a better outcome of 65.8 months (95% CI, 54.4 to not calculable) than the placebo (21.9 months—95% CI, 16.6 to 27.5), with a disease-free survival hazard ratio in favour of osimertinib of 0.23 (95% CI, 0.18 to 0.30). The authors reported a disease-free survival rate at 48 months of 70% (95% CI, 62 to 76) for osimertinib-treated patients versus a rate of only 29% (95% CI, 23 to 35) for patients who received only a placebo. Regarding the secondary endpoint, i.e., disease-free survival in the overall population of 682 patients with stage IB-IIIA disease, the outcome favoured osimertinib, with a hazard ratio of 0.27 (95% CI, 0.21 to 0.34), and with an impressive median disease-free survival of 65.8 months (95% CI, 61.7 to NC) for patients treated with adjuvant osimertinib versus only 28.1 months (95% CI, 22.1 to 35.0) for patients assigned to the placebo. Also, the disease-free survival rate at 4 years in the overall population favoured patients treated with adjuvant osimertinib, with this treatment arm achieving 73% (95% CI, 67 to 78) versus 38% (95% CI, 32 to 43) in the placebo arm. It should be underlined that this meaningful significant disease-free survival benefit achieved with adjuvant osimertinib was reported in all the patient subgroups, including patients who did and did not receive prior adjuvant chemotherapy, young and elderly patients, Asian and non-Asian patients, never smokers and smokers, and as reported before, in patients with any disease stage (IB, II and IIIA). For example, among patients with stage IB disease, the 4-year disease-free survival rates were 80% for osimertinib and 59% for the placebo, with a hazard ratio of 0.41 (95% CI, 0.23 to 0.69). Among those with stage II disease, these rates were 74% and 42%, with a hazard ratio of 0.34 (95% CI, 0.23 to 0.52), and among those with stage IIIA disease, the rates were 65% and 14%, with a hazard ratio of 0.20 (95% CI, 0.14 to 0.29). Another relevant topic emerging from the ADAURA trial is the site of recurrence, and specifically, the protection offered by osimertinib against the central nervous system disease recurrence. In patients with stage II-IIIA disease, central nervous system disease-free survival events were reported to be significantly less frequent in patients treated with osimertinib compared with patients assigned to the placebo, occurring in 9% and 17% of patients, respectively, with a hazard ratio favouring osimertinib of 0.24 (95% CI, 0.14 to 0.42). The protective effect of osimertinib against central nervous system recurrence was also substantial in the overall population (stage IB-IIIA disease), with an impressive disease-free survival hazard ratio of 0.36 (95% CI, 0.23 to 0.57). In the ADAURA trial survival outcomes were also recently reported, giving the complete and definitive crowning to osimertinib as the best and main adjuvant treatment for patients with resected EGFR-mutated NSCLC. Regarding the enrolled patients with stage II to IIIA disease, osimertinib achieved a 5-year overall survival rate of 85%, compared with 73% reported for patients treated only with the placebo (overall hazard ratio for death, 0.49; 95.03% confidence interval [CI], 0.33 to 0.73; *p* < 0.001). The improvement in survival rate was also reported in the overall population, thus extending also to patients with stage IB disease, with osimertinib-treated patients achieving a 5-year overall survival rate of 88% against a 78% rate for patients randomized to the placebo (overall hazard ratio for death, 0.49; 95.03% CI, 0.34 to 0.70; *p* < 0.001).

### 4.2. Safety Profile of Adjuvant Osimertinib, and Paradigm Shift from Toxic Chemotherapy

The safety profile of adjuvant osimertinib is to be considered very satisfactory for patients and physicians. In fact, in the ADAURA trial, the incidence of serious adverse events was globally low, and there was a modest difference among patients treated with osimertinib (20%) compared with those randomized to the placebo (14%). The only adverse event leading to death in the osimertinib group was not caused by the experimental drug. Also, the safety parameters, such as dose interruptions, dose reductions, and discontinuations of osimertinib due to toxic events were low (27%, 12%, and 13% versus 13%, 1%, and 3% for the placebo). The most frequent adverse events reported with osimertinib were diarrhea (47% versus 20% for the placebo), paronychia (27% versus 1% for the placebo), and dry skin (25% versus 7% for the placebo). Interstitial lung disease was reported more frequently in patients treated with osimertinib (3% against 0% of patients treated with the placebo), but this type of pulmonary toxicity was always mild (grades 1 or 2) and never fatal. Cardiac toxicity was very rare, being reported in 6% of patients treated with osimertinib and in 3% of patients treated with the placebo. Dermatitis acneiform was reported in 12% of patients treated with osimertinib, but these reports were only of grade 1 and 2. Nausea was reported in 10% of patients, but mainly (8%) of reports were grade 1. The paradigm shift, in terms of the safety profile, the quality of life and global treatment perception, from adjuvant chemotherapy to adjuvant osimertinib is deep and epochal. For the first time, for adjuvant treatment of resected lung cancer, we have something similar to adjuvant hormonal therapy for breast cancer, from both a patient’s and oncologist’s point of view. Physicians and patients both perceive the difference between adjuvant treatment with osimertinib alone and adjuvant chemotherapy as similar to the difference between having or not having experienced cancer disease. This difference is also due to the fact that the most popular and studied adjuvant chemotherapy is cisplatin vinorelbine, which is commonly associated with a severe safety profile and bad quality of life during the treatment period.

### 4.3. Adjuvant Chemotherapy Use before Osimertinib

Another very important topic analyzed by the ADAURA trial was the description of adjuvant chemotherapy administration and its relationship with osimertinib outcomes reported by Wu et al. [10]. It should be remembered that in the ADAURA trial, adjuvant chemotherapy was not mandatory and the choice to administer it before adjuvant osimertinib or the placebo was left to the oncologists together with their patients, as in current clinical practice. However, disease-free survival in the overall population (IB-IIIA), with and without adjuvant chemotherapy, was a pre-specified analysis. Moreover, the administration of adjuvant chemotherapy was described in terms of patient age, disease stage, and geographic location. Overall, the majority of patients enrolled in the ADAURA trial (60%), were administered with adjuvant chemotherapy before osimertinib or the placebo. As expected, the administration of adjuvant chemotherapy was more frequent in young patients (aged less than 70 years) (66%) compared with elderly patients (aged more than or equal to 70 years) (42%). As expected, and consistent with current clinical practice, oncologists and their patients preferred to use adjuvant chemotherapy more frequently in more advanced disease stages (76% in stages II to IIIA versus 26% in stage IB). On the contrary, it is not intuitively clear what the reasons were for the more frequent use of adjuvant chemotherapy among Asian patients (65%) versus non-Asian patients (53%). A very relevant result of this pre-specified analysis of the ADAURA trial is that the administration of chemotherapy did not influence the occurrence or the magnitude of the benefit given by osimertinib administration. In fact, a disease-free survival benefit in favour of osimertinib versus the placebo was reported both for patients treated also with chemotherapy (DFS hazard ratio = 0.16, 95% confidence interval: 0.10–0.26) and for those treated without adjuvant chemotherapy (hazard ratio = 0.23, 95% confidence interval: 0.13–0.40). Moreover, this equivalence of effect was reported in all disease stages.

### 4.4. Summary of ADAURA Trial Outcomes

The above-mentioned data, taken together, definitively demonstrate adjuvant treatment with osimertinib for three years as a very effective treatment for patients with resected stages IB to IIIA EGFR-mutated NSCLC. This benefit is offered by osimertinib treatment both for patients treated with previous adjuvant chemotherapy and those not treated, even if in the ADAURA trial, as in current clinical practice, the administration of adjuvant chemotherapy before osimertinib has occurred more frequently in young patients and in higher stages of disease. In conclusion, the efficacy outcomes improved by adjuvant osimertinib in the ADAURA trial and offered to patients with resected EGFR-mutated stage IB-IIIA NSCLC are a statistically but also clinically significant prolongation of disease-free survival and overall survival. They also represent a reduced risk of local and distant recurrence, a protective effect against central nervous system involvement, and last but not least, the treatment has an excellent safety profile during the three-year treatment period.

## 5. Shared Decision Making When Administering Adjuvant Chemotherapy to Patients Whose Tumors Harbour EGFR Mutation

Considering the above-mentioned recent data from the ADAURA trial, osimertinib has been reported to achieve a 5-year overall survival rate of 85% for patients with stage II to IIIA resected NSCLC harbouring EGFR mutations against the 73% achieved by placebo. Thus, in a clinical condition where adjuvant cisplatin-based chemotherapy may be proposed, osimertinib offers a clinically relevant absolute risk reduction in death of 12% with a low number needed to treat of 8. Thus, we may consider with our patients the idea of administering additional toxic chemotherapy with a further theoretical ARR of 4% and NNT of 25 or to remain satisfied with the great benefit offered by osimertinib alone. But, is the benefit offered by chemotherapy in EGFR-mutated patients treated also with adjuvant osimertinib separate and unaltered or does the benefit partially overlap with that of osimertinib and further reduce in this patient population? And, moreover, does the delay in the beginning of adjuvant osimertinib due to adjuvant chemotherapy impair the benefit of osimertinib? We have no data to answer to these questions. In our opinion, in EGFR-mutated NSCLC patients, given the outcomes of adjuvant chemotherapy and of adjuvant osimertinib, the option of administering chemotherapy should be restricted only to young and fit patients. In this patient population, detailed communication and explanation of the absolute benefit of adding chemotherapy to osimertinib should occur, and chemotherapy should not be denied to conscious and willing patients. In elderly and/or unfit patients with comorbidities, when only sub-optimal and non-cisplatin-containing chemotherapy could be administered, chemotherapy should be omitted and osimertinib directly administered (Table 1).

## 6. Adjuvant Chemotherapy in ALK-Rearranged Resected NSCLC

Alectinib is the first ALK inhibitor that has been tested in the adjuvant context of NSCLC. In a phase III randomized trail named ALINA, recently presented at the ESMO Congress 2023, 253 patients with stage IB-IIIA resected ALK-positive NSCLC were randomized to receive either oral alectinib 600 mg twice daily, or four cycles of platinum-based adjuvant chemotherapy [22]. Alectinib was given for up to 24 months or until disease recurrence or unacceptable toxicity. The primary endpoint was disease-free survival, tested hierarchically first in the stage II–IIIA and then in the intention-to-treat population (stage IB–IIIA). A significant disease-free survival benefit was reported in favour of alectinib versus adjuvant chemotherapy in both the stage II-IIIA (HR 0.24; 95% CI: 0.13–0.45; *p* < 0.0001) and intention-to-treat populations (HR 0.24; 95% CI: 0.13–0.43; *p* < 0.0001), also with a pronounced protective effect offered by alectinib against central nervous system involvement. The magnitude of the disease-free survival benefit may also translate into survival benefit, and overall survival data from the ALINA trial, immature at the time of this analysis, are eagerly awaited. In terms of two-year disease-free survival rates, alectinib was demonstrated to be clearly superior compared with chemotherapy in patients with stage II-IIIA resected disease, achieving a rate of 93.8% versus the rate of 63.0% achieved by chemotherapy. Also, in terms of safety profile, the outcomes of alectinib were satisfactory. In fact, the rates of grade 3–4 adverse events were similar between the two strategies (30% for patients administered with alectinib and 31% for those administered with chemotherapy). Likewise, the rates of serious adverse events (13% of patients treated with alectinib and 8% of those treated with chemotherapy) were also similar. In contrast, the rates of serious treatment-related adverse events and adverse events leading to treatment withdrawal favoured alectinib. In fact, for patients treated with the ALK inhibitor, the rates were 2% and 5% respectively, while for patients treated with chemotherapy, the rates were 7% and 13%, respectively. In our opinion, the safety data of alectinib as adjuvant therapy are satisfactory but not as good as those of osimertinib. However, the magnitude of the clinical benefit offered by adjuvant alectinib appear to be really impressive in terms of disease-free survival benefit across all stages of resected disease and in terms of central nervous system protection. Thus, although overall survival data are not yet available, the outcomes from the ALINA trial on adjuvant alectinib in patients with ALK-positive resected NSCLC clearly appear to be practice-changing. Another important point that makes the results of the ALINA trial of particular relevance is the decision of the investigators to compare the ALK-inhibitor and chemotherapy directly (not ALK-inhibitor added after chemotherapy and placebo, as for the EGFR inhibitor osimertinib in the ADAURA trial). In our opinion this decision, given the great clinical effect of alectinib, has been successful because it gives physicians and patients, both a new and more effective treatment option and the definitive omission of chemotherapy in this patient population. Thus, in patients with resected ALK-positive NSCLC, the difficult decision to administer chemotherapy or not before the targeted agent, as for patients with EGFR-mutated NSCLC, can be omitted.

## 7. Adjuvant Chemotherapy in the New Era of Adjuvant Immunotherapy

The role of adjuvant chemotherapy has to be rediscussed, mainly in relation to non-oncogene-addicted disease, after the outcomes recently reported with adjuvant immunotherapy. In the phase III randomized trial named Impower010, a statistically and clinically significant disease-free survival benefit was demonstrated in favour of adjuvant immunotherapy versus best supportive care (both after adjuvant chemotherapy) in patients with resected NSCLC [23,24]. The authors of the IMpower010 trial randomized about 1000 patients with resected stage IB (≥4 cm) -IIIA NSCLC to receive adjuvant immunotherapy with the anti-programmed death-ligand 1 (PDL-1) monoclonal antibody atezolizumab (1200 mg every 3 weeks for 16 cycles) or best supportive care after adjuvant platinum-based chemotherapy up to 4 cycles. In patients with stage II-IIIA NSCLC whose tumors expressed PD-L1 on ≥1% of tumor cells (PD-L1 TC ≥ 1%), the hazard ratio for DFS was 0.66 [95% confidence interval (CI) 0.50–0.88], while in patients with PD-L1 TC ≥ 50%, the resulting benefit was even greater, with a hazard ratio for DFS of 0.43 (95% CI 0.27–0.68). Moreover, adjuvant immunotherapy with atezolizumab was also reported to improve the overall survival of patients whose stage II-IIIA tumors expressed PDL-1 on ≥50% of tumor cells (exploratory analysis of a secondary endpoint), with a hazard ratio of 0.43 (95% CI 0.24–0.78). Also, the safety profile of adjuvant atezolizumab was considered mild, with severe adverse events (≥grade 3) being reported in about 11% of patients. The relevant clinical data from the IMpower010 trial have led to adjuvant atezolizumab being introduced as a standard of care in clinical practice, but with some particularities in different countries. For example, in the United Stated, adjuvant atezolizumab is approved after adjuvant platinum-based chemotherapy for all patients whose resected stage II-IIIA tumors express PD-L1 on ≥1% of tumor cells, while oncologists in the European Union are authorized to administer adjuvant atezolizumab after chemotherapy only in patients whose resected stage II-IIIA tumors express PD-L1 on ≥50% of tumor cells stage (with the exclusion of patients whose tumors harbour EGFR mutations and ALK rearrangements), and always after chemotherapy. These atezolizumab outcomes in the adjuvant setting, in our opinion, strengthen the role of adjuvant chemotherapy in non-oncogene-addicted resected NSCLC when immunotherapy can be administered because chemotherapy (even if sub-optimal for advanced age and/or comorbidities) is often seen by physicians and patients also as a road or a bridge to administer immunotherapy. Also, the anti PDL-1 monoclonal antibody pembrolizumab has recently been adopted in the clinical practice of adjuvant strategies of resected NSCLC, after adjuvant chemotherapy or directly after surgery, as a unique treatment. In the phase III randomized trial named PEARLS/KEYNOTE-091, the investigators randomized 1177 patients with resected stage IB-IIIA NSCLC with any level of PD-L1 expression, including <1%, to receive pembrolizumab at the dose of 200 mg or a placebo every 21 days for a maximum of 18 cycles unless progressive disease or excessive toxicity occurred [25]. In this trial, the investigators were not obliged to administer chemotherapy. It was, however, considered as an option for patients with stage IB disease and, on the contrary, recommended for patients with higher disease stages, i.e., stage II and IIIA. In the overall population of the trial, for patients whose tumors expressed PDL-1 at any level, pembrolizumab achieved a better result than the placebo, with a median disease-free survival of 53·6 months (95% CI 39.2 to not reached) versus 42.0 months (31.3 to not reached) of placebo (HR 0.76 [95% CI 0.63–0.91], *p* = 0.0014). In contrast, in the other analyzed population, i.e., patients whose tumors expressed PD-L1 with a tumor proportion score equal to or greater than 50%, pembrolizumab achieved similar outcomes compared with the placebo, with a median disease-free survival not reached in either the pembrolizumab group (95% CI 44.3 to not reached) or the placebo group (95% CI 35.8 to not reached; HR 0.82 [95% CI 0.57–1.18]; *p* = 0.14). The reported disease-free survival outcomes were the primary endpoints of the trial. Also, the safety profile of pembrolizumab was confirmed to be satisfactory, with serious adverse events occurring in 24% of patients. As a consequence of the results from the PEARLS/KEYNOTE-091 trial, pembrolizumab has been recently approved for use in the United States and the European Union as an adjuvant treatment option for patients with resected stage IB-IIIA NSCLC, with or without previous chemotherapy and with any PDL-1 expression. Even if it not mandatory to link adjuvant pembrolizumab administration with chemotherapy, in our opinion, its use will not limit the administration of osimertinib chemotherapy in EGFR-mutated disease. On the contrary, the outcomes of adjuvant pembrolizumab are not comparable in terms of the magnitude of clinical benefit with those of adjuvant osimertinib, making its utilization synergistic with adjuvant chemotherapy and thus reinforcing the role of adjuvant chemotherapy in EGFR WT resected NSCLC (Table 2). However, the role of adjuvant immunotherapy in resected non-oncogene-addicted NSCLC should be rediscussed, considering also the relevant recent data for the neoadjuvant and perioperative combination of chemotherapy and immunotherapy [26]. On the contrary, the role of targeted agents such as osimertinib and alectinib in a neoadjuvant approach in oncogene-addicted resectable NSCLC is yet to be defined, with phase III trials ongoing [27], and with the adjuvant approach to be considered the only standard of care in the near future.

## 8. Conclusions

The decision to administer or not administer adjuvant medical treatment to patients with resected cancer is always challenging, but in some clinical conditions, as with resected NSCLC, the risk–benefit ratio is particularly border-line and explaining it to patients is extremely difficult. For patients with EGFR-mutated resected NSCLC, a new, more effective, and better tolerated treatment option exists with oral osimertinib, making for this patient population the role of adjuvant chemotherapy less relevant and also more difficult to explain and comprehend. Based on the data and considerations presented above, in our opinion, adjuvant chemotherapy before osimertinib in patients with resected NSCLC harbouring EGFR mutations should be omitted in elderly patients (over 70 years) and in all patients with comorbidities that would increase the risk of death during chemotherapy or that would prevent physicians from the administration of optimal cisplatin-based chemotherapy. However, the possibility of administering chemotherapy should always be shared with eligible patients, and this treatment option should not be denied to willing patents. Young and fit patients should also have the option to omit chemotherapy and be administered adjuvant osimertinib directly if this is their preferred shared treatment strategy. It should be underlined that the idea to share with patients with EGFR-mutated resected NSCLC the possibility of being administered adjuvant chemotherapy before osimertinib only if “young” and “fit” and not if “elderly” and or “with relevant comorbidities” cannot be considered as completely evidence-based, but may be a “good-sense” decision based on a global view of all the above-mentioned data. The novel aspect of this paper is to suggest a communication approach which stimulates and improves shared decision making when deciding about adjuvant therapies for NSCLC. Specifically, when communicating to resected EGFR-mutated NSCLC patients about the possibility of adjuvant chemotherapy before osimertinib, the main points to highlight and discuss with them are as follows: the approximate recurrence rate of disease, the absolute reduction in risk of death given by osimertinib alone, the further presumed absolute risk reduction given by the administration of chemotherapy before osimertinib, the risk of severe adverse events correlated with chemotherapy, and the impact on quality of life of chemotherapy. The available scientific data suggest that the contribution of adjuvant chemotherapy to reducing the risk of death of resected EGFR-mutated NSCLC patients is really limited, and the treatment is associated with a non-negligible risk of severe adverse events. However, fit and young patients, who after receiving explanations of such limited and costly to achieve benefits, express the willingness to undergo adjuvant therapies to reduce their presumed absolute risk of death by one or two percentage points, should be supported in their choice, and administered the best cisplatin-based chemotherapy with a pro-active approach for the prevention of severe adverse events.

## Figures and Tables

**Table 1:** Absolute risk reduction (ARR) of death at 5 years, number needed to treat (NNT) to achieve the outcome (patient alive at 5 years) and risk of severe adverse events (≥grade 3) with adjuvant chemotherapy and adjuvant osimertinib.

	ARR	NNT	SAE
Adjuvant cisplatin-based chemotherapy in EGFR WT NSCLC	4	25	80% [4] (cisplatin-vinorelbine)
Adjuvant osimertinib in EGFR-mutated NSCLC	12	8	23% [9]
Adjuvant cisplatin-based chemotherapy added to osimertinib in EGFR-mutated NSCLC	4 or less?	25 or more?	80% [4] (cisplatin-vinorelbine)

**Table 2:** Main outcomes of adjuvant immunotherapy and adjuvant alectinib in resected NSCLC.

	Disease-Free Survival Advantage versus Placebo or Chemotherapy (Hazard Ratio)	Serious Adverse Events
Atezolizumab after chemotherapy versus placebo PDL-1 > 1% [23,24]	0.66 (95% CI 0.50–0.88)	11%
Atezolizumab after chemotherapy versus placebo PDL-1 > 50% [23,24]	0.43 (95% CI 0.27–0.68)	11%
Pembrolizumab after chemotherapy or alone versus placebo Any PDL-1 expression [25]	0.76 (95% CI 0.27–0.91)	24%
Pembrolizumab after chemotherapy or alone versus placebo PDL-1 > 50% [25]	0.82 (95% CI 0.57–1.18)	24%
Alectinib versus chemotherapy [22]	0.24 (95% CI 0.13–0.45)	13%

## Data Availability

Not applicable.
